# Quantifying Contextual Information For Cognitive Control

**DOI:** 10.3389/fpsyg.2018.01693

**Published:** 2018-09-10

**Authors:** Francisco Barceló, Patrick S. Cooper

**Affiliations:** ^1^Laboratory of Neuropsychology, University of the Balearic Islands, Mallorca, Spain; ^2^Functional Neuroimaging Laboratory, School of Psychology, University of Newcastle, Newcastle, NSW, Australia; ^3^Priority Research Centre for Stroke and Brain Injury, University of Newcastle, Newcastle, NSW, Australia; ^4^Priority Research Centre for Brain and Mental Health, University of Newcastle, Newcastle, NSW, Australia

**Keywords:** information theory, Bayesian inference, context-updating, context-learning, frontoparietal cortical dynamics, P300

## Introduction

**Figure 1:**
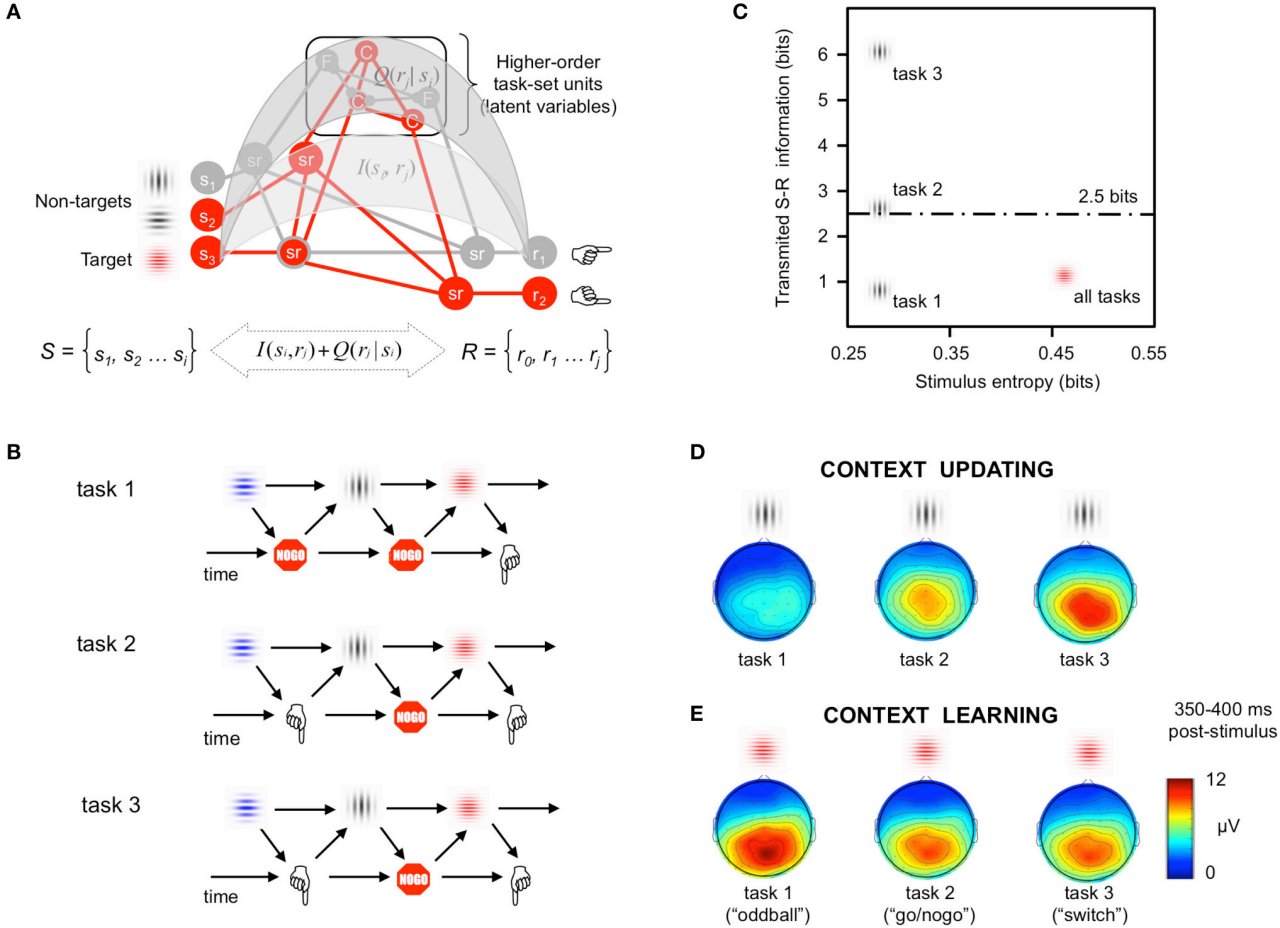
Formal modeling of contextual information. **(A)** Hierarchies of cognitive control. Information theory can be used to quantify the contextual dependencies characterizing cognitive control in simple target detection tasks, as well as in more complex tasks involving hypothetical high-order latent variables (here, Color and Form rules). Mean probability of task events (i.e., *P* = *0.2* for gray non-targets, *P* = *0.8* for colored targets) cannot fully convey the complex contextual contingencies driving behavioral and brain responses in studies on cognition. Information theory metrics such as stimulus entropy, and information transmission between sets of task stimuli (*S*) and responses (*R*)–both at lower [*I*(*s*_*i*_, *r*_*j*_)] and higher [*Q*(*r*_*j*_ *|s*_*i*_)] ordered levels in the neural hierarchy of control (Koechlin and Summerfield, 2007), offer better ways to parametrize the numerous sources of contextual information that modulate behavioral and brain responses in studies of cognition (adapted by permission from, Miller, 2000). **(B)** Time dynamics of sensorimotor loops. Examples of three cognitive tasks where the stimulus context was kept constant while manipulating motor and sensorimotor demands (Barcelo and Cooper, 2018). Task 1 (“*oddball task*”) involved detection of visual targets using one-forced choice responses (“press a button to red gratings”); Task 2 (“*go/nogo task*”) required two-forced choice responses (“press button 1 for red gratings and button 2 for blue gratings”); In Task 3 (“*switch task*”) infrequent vertical and horizontal gray gratings instructed participants to switch and repeat the active task rule (i.e., “Color” vs. “spatial Frequency”), respectively. **(C)** Quantifying contextual information. Transmitted sensorimotor (S-R) information was modeled at two levels in the hypothetical neural hierarchy shown in **(A)**, and plotted as a function of mean stimulus entropy (Miller, 1956). This simple model predicted maximal task differences in contextual information among the temporarily surprising non-target stimuli, and no differences in task-averaged transmitted information for the temporarily predictable target stimuli (adapted by permission from, Barcelo and Cooper, 2018). **(D)** Context updating: Scalp-recorded “context P3” responses to the surprising non-target “nogo” stimuli (300–450 ms poststimulus) captured the graded differences in cognitive demands across all three tasks, as predicted by the model in **(C)**. The largest “context P3” intensities were observed in the task with the largest sensorimotor entropy, a condition conveying maximal contextual uncertainty about upcoming actions (adapted by permission from, Barcelo and Cooper, 2018). Similar context-sensitive brain responses have also been reported when using auditory and somatosensory stimulation (Donchin, 1981). **(E)** Context learning: The intensity of “target P3” responses to temporarily predictable target “go” stimuli was slightly larger in the task conveying less sensorimotor entropy, whose contextual information could be quickly learned (adapted by permission from Wiley: Barcelo and Cooper, 2018). These findings pointed to a common fronto-parietal cortical network for cognitive control showing different functional dynamics during two temporarily distinct context updating and context learning stages of processing.

In the tradition of serial processing models that link the stimuli, the organism and its responses (S → O → R) (Hull, 1943), cognitive control has long been defined from stimulus-centric views implicit in simple “target detection” tasks (e.g., flanker and Stroop-type tasks) (Posner and Petersen, 1990). According to these views, cognitive control is invoked for processing (i.e., perceiving, attending, memorizing) and, ultimately responding to, a task-relevant set of stimulus features. These task-relevant features occur in close spatiotemporal proximity to competing non-targets and distracters that need not be fully processed or responded to. As a consequence, cognitive demands are normally defined as a function of such sets of relevant and irrelevant stimulus features. These features compete for limited central resources at the cost of less efficient goal-directed behavior. However, in such stimulus-centric views, the actions themselves are largely disregarded. For example, a button press is simply considered the final output of stimulus-feature competition with no or little influence on earlier information processes. Such stimulus-centric views permeate interpretation of other executive function tasks like the Wisconsin card sorting test (WCST), and task-switching paradigms, inasmuch as these more complex tasks share common features with simpler target detection paradigms. Indeed, in all these tasks, targeted stimulus features appear within the spatiotemporal context of other nontarget features that may afford alternative and potentially conflicting courses of action. Here we embrace current enactivist views of cognition to propose an alternative framework to probe cognitive control. To do so, we contend that (1) cognitive control is essentially *context-sensitive*, and hence (2) cognitive demands should be defined and quantified in terms of *contextual information or uncertainty*. We suggest shifting the focus of context to specifically capture the uncertainty about upcoming actions, which may be represented in the brain through hierarchies of sensorimotor loops that evolve dynamically over time (Figures 1A,B; cf., Miller, 2000; Fuster, 2001; Kilner et al., 2015; Friston et al., 2017).

## Beyond stimulus-centric views of cognitive control

Enactivist views of cognition bring the focus onto perception-action cycles (or sensoriomotor loops) that link the external and internal worlds, and explain how organisms adapt to their environment in a context-specific manner (see the new action-oriented version of the Bayesian brain hypothesis, Friston et al., 2017). From such views, cognitive control of behavior involves the preparation and execution of stimulus-response (S-R) mappings (perception-action rules), that link sensory evidence to actions in a dynamic and context-sensitive way (cf., Figure 10.2 in Kilner et al., 2015). As stressed earlier, this involves not only the sensory stimuli but also, critically, their probabilistic association with available actions and potential outcomes. While the associations between a stimulus set and potential actions can be created in multifaceted ways in cognitive tasks—either through habit, learning, or made explicit with task instructions—once established, these task sets need to be held in active memory and updated on a trial by trial basis for efficient goal-directed behavior (Figure 1A, cf., Miller, 2000; Miller and Cohen, 2001). Here we posit that all these elements of a task set ought to be incorporated into more comprehensive formal models of higher cognition (Boureau et al., 2015). To do so, we can use probability theory tools, such as *Bayesian statistics* and *Information theory* metrics (e.g., stimulus and response entropies, information transmission, etc.), to quantify contextual information as numerical regressors for modeling task-averaged behavioral and brain responses and their trial-by-trial dynamics (Koechlin and Summerfield, 2007; Friston et al., 2017).

Modern enactivist views portray cognition in terms of dynamic contextual contingencies of an agent's brain with internal states, whose function is to act on an environment containing many external states that remain hidden behind sensory states. Internal states refer to any neural activities, connection strengths, etc., characterizing the brain at one point in time. External states cause sensory states which in turn change internal states. Conversely, internal states cause changes in agential states (e.g., muscles), which in turn change external states. According to this view, sensory and agential states link the world to the brain through perception-action cycles (i.e., sensorimotor loops) that evolve dynamically with time (cf., Figure 10.2 in Kilner et al., 2015). Under the assumption that such contextual dependencies obey probability laws, they can be formally quantified with Bayesian statistics and Information theory.

## Time dynamics of perception-action cycles

From enactivist views of cognition, information processing can be described in terms of cyclic, time dependent statistical regularities between task stimuli (*s*) and associated actions and/or responses (r) that evolve dynamically over time (Figures 1A,B). This schematic highlights the key role of the contextual contingencies when characterizing information processing in the brain (Kilner et al., 2015; Friston et al., 2017). Contextual information in cognitive tasks (lines and arrows in Figures 1A,B) can be quantified in terms of statistical regularities among sensory inputs, motor outputs (actions), and any intermediate (hidden) processes characterizing external and internal states of the acting agent. When used as predictors of behavioral and brain responses, numerical estimates of contextual information are more amenable to modeling and falsification than traditional taxonomies based on stimulus categories (i.e., relevant target vs. irrelevant distractor), or instructed task conditions (i.e., switch vs. repeat cues) (Barcelo and Cooper, 2018). Here we want to emphasize the temporal dynamics and statistical regularities (denoted by the grid of lines and arrows in Figures 1A,B) between stimuli and their associated actions and outcomes, as key elements to estimate information processes in a cognitive task, over and above the specific physical content and modality of sensory afferents and agential effectors (denoted as stimulus and response illustrations in Figures 1A,B).

One recent study attests to the importance of using numerical estimates of contextual information instead of nominal stimulus taxonomies to clarify the complex neural dynamics underlying cognitive control (Barcelo and Cooper, 2018). This study used exactly the same sequence of visual stimuli under slightly different response demands (Figure 1B). Temporarily unpredictable gray gratings that were not to be responded to (nontarget “nogo” stimuli) set the temporal context for responding to the ensuing and temporarily predictable red colored gratings (target “go” stimuli) in all three tasks. Importantly, whereas in tasks 1 and 2 the explicit S-R mapping between colored gratings and motor actions was held constant throughout (thus favoring “context learning”), in task 3 the S-R mapping was to be either intermittently switched (prompting for “context updating”), or kept constant during the following trial run, depending on the orientation of the gray gratings, respectively (Barcelo and Cooper, 2018).

This study identified functionally distinct neural signatures for two different sources of contextual uncertainty, namely, temporal and task uncertainly. The *temporal uncertainty* conveyed by the temporarily unexpected nontarget stimuli had a graded impact on both behavioral costs and associated brain responses (Figures 1D,E), despite similar sensory inputs used in all task conditions, and regardless of the instructed nominal meaning of those nontarget stimuli. Thus, resolution of temporal uncertainty about *when* to implement an action plan was one key source of behavioral and neural variability that did not readily transpire from stimulus definitions alone. Instead, temporal uncertainty was more accurately estimated as a function of stimulus entropy (Figure 1C). Likewise, resolution of *task uncertainty* about *what* was to be done with target stimuli (e.g., either to sort them by color only in tasks 1 and 2, or to alternate between color and form rules in task 3) gradually modulated behavioral and brain responses. Task uncertainty was numerically estimated as a function of the sensorimotor entropy in specific task conditions (Barcelo and Cooper, 2018). The schematic in Figures 1C shows how these two sources of contextual information (temporal and task uncertainty) can be numerically quantified and linked with cognitive demands. This study exemplifies how quantification of contextual information in terms of time-dependent statistical regularities among the sensory, motor and sensorimotor elements of a cognitive task, is critical for a meaningful characterization of cognitive brain responses over and above traditional nominal stimulus taxonomies alone (cf., also Koechlin and Summerfield, 2007; Friston et al., 2017).

## Context updating versus context learning

Numerical estimations of contextual information may help elucidate long-standing issues in cognitive neuroscience that have historically been interpreted within nominal stimulus taxonomies. A salient example is the so-called “P300” (or P3) component of the human brain potential, one of the most widely employed neural indexes of cognition. After long-lived controversies about its function, the parietal aspect of P3 has been linked to the updating of contextual information in response to surprising target stimuli (Donchin, 1981), as well as to the gradual accumulation of sensory evidence (Twomey et al., 2015). However, these stimulus-centric views of P3 function fail to offer an overarching account of its complex functional diversity and varying fronto-parietal scalp distribution in response to both target and nontarget stimuli in many task domains. To address this, Barcelo and Cooper (2018) relied on numerical estimates of contextual information to identify two broad families of functionally distinct P3-like signals whose intensities and fronto-parietal scalp distributions varied parametrically as a function of the amount of sensory and sensorimotor entropy conveyed by the eliciting stimulus (Figures 1D,E). Importantly, these P3 signals tracked the interaction between temporal and task uncertainty at the onset of nontarget “nogo” stimuli that anticipated upcoming target “go” stimuli disclosing the remaining information necessary to decide upon one of two possible actions. The rich parametric modulations of P3-like potentials spoke of correspondingly complex functional dynamics of a common frontoparietal network for cognitive control that was putatively engaged during both context-updating and context-learning operations to non-target “nogo” gray stimuli and target “go” colored stimuli, respectively, in line with numerical estimates of contextual information (Figures 1C–E), albeit with very different functional roles in each case (i.e., context updating preceded in time and seemed a prerequisite for context learning). These findings were compatible with claims about two complementary modes of cognitive control, which have been variously named as proactive and reactive, exploration and exploitation, or context updating and context learning (Braver, 2012; Boureau et al., 2015; Friston et al., 2017; Barcelo and Cooper, 2018).

## Conclusion

Cognitive demands can be numerically quantified as contextual information using Information theory and Bayesian statistics. Contextual information modulates both behavioral and brain responses in cognitive control tasks. Thus, it has been recently shown that the temporal context yields exploratory (proactive) and exploitative (reactive) control modes (Barcelo and Cooper, 2018). Moreover, context updating precedes and is a prerequisite for context learning in two-modes models of cognitive control (Friston et al., 2017). In sum, here we submit that quantifying contextual information can foster progress on human cognition and psychiatric research (Silverstein et al., 2017), it blends well with modern views of cognitive control as associative learning (Abrahamse et al., 2016), and with the human connectome (Sporns, 2011), and provides researchers with a common metric to compare cognitive demands across many different task domains (Miller, 1956; Duncan, 2010).

## Author contributions

Both authors conceived this opinion paper. FB wrote the first draft of the manuscript. Both authors contributed to manuscript revision, read, and approved the submitted version.

### Conflict of interest statement

The authors declare that the research was conducted in the absence of any commercial or financial relationships that could be construed as a potential conflict of interest.
